# CircDNA2‐Educated YTHDF2 Phase Separation Promotes PM_2.5_‐Induced Malignant Transformation Through the Blunting of GADD45A Expression

**DOI:** 10.1002/advs.202410532

**Published:** 2025-01-17

**Authors:** Jie Xu, Zhi Ling, Lijia Yin, Duo Xu, Shenshen Wu, Rui Chen

**Affiliations:** ^1^ Yunnan Provincial Key Laboratory of Public Health and Biosafety & School of Public Health Kunming Medical University Kunming 650500 P. R. China; ^2^ School of Public Health Capital Medical University Beijing 100069 P. R. China; ^3^ Laboratory for Environmental Health and Allergic Nasal Diseases Laboratory for Clinical Medicine Capital Medical University Beijing 100069 P. R. China; ^4^ Beijing Laboratory of Allergic Diseases Capital Medical University Beijing 100069 P. R. China; ^5^ Department of Occupational and Environmental Health Fourth Military Medical University Ministry of Education Key Lab of Hazard Assessment and Control in Special Operational Environment Xi'an 710032 P. R. China

**Keywords:** circrna, liquid–liquid phase separation, malignant transformation, PM2.5, YTHDF2

## Abstract

Substantial epidemiological evidence suggests a significant correlation between particulate matter 2.5 (PM_2.5_) and lung cancer. However, the mechanism underlying this association needs to be further elucidated. Circular RNAs (circRNAs) have emerged as an important topic in the field of epigenetics and are involved in various cancers. This study aimed to explore the molecular basis of PM_2.5_‐induced lung cancer from an epigenetic perspective and identify potential biomarkers. Initially, the construction of a chronic PM_2.5_ exposure model confirmed that PM_2.5_ exposure promoted the malignant transformation of human bronchial epithelial (HBE) cells. Mechanistically, abnormally upregulated circDNA2 inhibited the tumor suppressor gene growth arrest and DNA damage 45 alpha (GADD45A) mRNA in an N6‐methyladenosine (m^6^A)‐dependent manner, mediated by YTH N6‐Methyladenosine RNA Binding Protein F2 (YTHDF2) after PM_2.5_ exposure. Further analyses revealed that circDNA2 can specifically bind to the YTHDF2 LC domain to promote YTHDF2 protein liquid–liquid phase separation (LLPS), providing sufficient evidence linking LLPS and particulate pollutant‐induced tumorigenesis. In conclusion, this study provides new insights into the role of circDNA2 in PM_2.5_‐induced lung cancer and confirms its clinical value as a potential prognostic biomarker for lung cancer.

## Graphical abstract

This study unveiled the mechanism by which circDNA2‐educated YTHDF2 phase separation promotes PM_2.5_‐induced malignant transformation in HBE cells by blunting GADD45A expression, providing definitive evidence for the first time that LLPS is involved in the induction of tumorigenesis by particulate pollutants. Moreover, the clinical value of circDNA2 and GADD45A as potential prognostic biomarkers of lung cancer are evaluated.

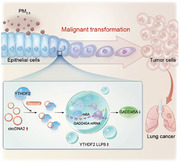

## 1 Introduction

Lung cancer is one of the most common malignancies and the leading cause of cancer‐related deaths, with an estimated 2.5 million new cancer cases and 1.8 million deaths in 2022.^[^ ^1^ ^]^ In addition to genetic and epigenetic alternations, environmental factors also contribute to lung cancer progression.^[^ ^2^ ^]^ Particulate matter 2.5 (PM_2.5_) is a class A carcinogen that is associated with an increased risk of lung cancer.^[^ ^3^ ^]^ A 5 µg m^−3^ increase in PM_2.5_ levels was associated with an increased hazard ratio of lung cancer incidence by 1.63 in a study conducted in the UK.^[^ ^2^ ^]^ A nationwide study conducted across 295 Chinese counties revealed that a 10 µg m^−^^3^ increase in PM_2.5_ exposure was associated with a 4.20% higher rate of lung cancer in males and a 2.48% increase in females.^[^ ^4^ ^]^ In 2019, household PM_2.5_ exposure was responsible for 0.08 million deaths and 1.94 million disability‐adjusted life‐years.^[^ ^5^ ^]^ Despite numerous epidemiological studies confirming that PM_2.5_ exposure is a risk factor for lung cancer, the molecular mechanisms underlying PM_2.5_‐induced lung cancer remain largely unknown.^[^ ^6^ ^]^

Circular RNAs (circRNAs) are a class of noncoding RNAs that are expressed in a tissue‐ and developmental stage‐specific manner. CircRNAs have been reported to regulate gene expression through various mechanisms, including acting as microRNA sponges, regulators of transcription and splicing, protein scaffolds, and regulators of translation.^[^ ^7^ ^]^ Accumulating studies have shown that circRNAs play a crucial role in lung cancer development and progression.^[^ ^7^, ^8^ ^]^ Moreover, circRNAs have also been reported to be involved in environmental pollutant‐induced cellular dysfunction.^[^ ^9^ ^]^ However, whether circRNAs contribute to PM_2.5_‐induced lung cancer remains to be elucidated. Therefore, this study focused on the role of circRNAs as potential mediators in this relationship.

Liquid–liquid phase separation (LLPS) is a focus of growing research interest because it plays an important role in cellular physiology and pathology. Normal phase separation is involved in the regulation of gene expression and signaling, whereas abnormal phase separation is associated with neurodegenerative diseases and cancer.^[^ ^10^ ^]^ CircRNAs have been shown to regulate cancer progression by participating in LLPS events.^[^ ^11^ ^]^ LLPS was incorporated into this study as an innovative means of exploring the regulatory pathways underlying particulate pollutant‐induced tumorigenicity, providing a new perspective for the exploration of the mechanisms underlying PM_2.5_‐induced lung cancer through the clarification of the association between circRNAs and LLPS.

In the present study, we found that PM_2.5_ can affect the regulatory network involving circDNA2, and ultimately accelerating the malignant transformation of lung epithelial cells and inducing lung cancer development. This study presents a novel mechanism by which circRNAs can influence PM_2.5_‐induced lung cancer and provides definitive evidence that LLPS is involved in the induction of tumorigenesis caused by particulate pollutants.

## 2 Results

### 2.1 Long‐Term PM_2.5_ Exposure Promotes the Malignant Transformation of HBE Cells

To examine the effect of particulate matter 2.5 (PM_2.5_) exposure on lung cancer, we explored tumorigenic phenotypes in lung epithelial cells by constructing a chronic PM_2.5_ exposure model (Figure S1A, Supporting Information). As depicted in Figure S1B–D, Supporting Information, compared with control cells, PM_2.5_ exposure markedly increased human bronchial epithelial (HBE) cell colony formation, migration, and invasion in a dose‐dependent manner. Furthermore, we established three murine models and found that long‐term PM_2.5_ exposure resulted in increased tumor growth and enhanced hepatic or lung metastasis (Figure S1E–G, Supporting Information). In conclusion, both in vivo and in vitro experimental results showed that chronic PM_2.5_ exposure played a key role in lung tumorigenesis.

### 2.2 Expression and Characteristics of circDNA2 in HBE Cells Subjected to Long‐Term PM_2.5_ Exposure

**Figure 1:**
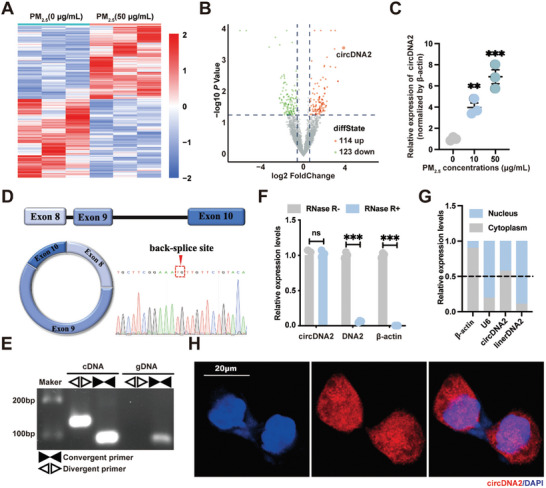
Expression and characteristics of circDNA2 in HBE cells subjected to long‐term PM_2.5_ exposure. A). Three pairs of control HBE and chronic PM_2.5_‐exposed HBE cells were subjected to circRNA‐seq assay. Heatmaps revealed differentially expressed circRNAs. B). The volcano plot showed the differentially expressed circRNAs based on circRNA‐seq data analysis. C). CircDNA2 mRNA levels in PM_2.5_‐treated cells (n = 3 per group, compared with the group with 0 µg mL^−1^ exposure concentration of PM_2.5_, one‐way ANOVA, error bars represent SD, duplicate measurement per replicate). D). The circDNA2 genomic locus. The unique backspliced junction fragment of circDNA2 was validated by Sanger sequencing. E). qRT‐PCR analysis of circRNAs amplified using divergent or convergent primers in HBE cells. F). circDNA2 expression in RNase R‐treated HBE cells (n = 3 per group, compared with the RNase R^−^ within each group, two‐tailed *t*‐test, error bars represent SD, duplicate measurement per replicate). G–H). The distribution of circDNA2 in HBE cells was detected by qRT‐PCR and RNA‐FISH. ns: no significant difference, ^**^ *p* < 0.01, ^***^ *p* < 0.001. Representative staining images are shown, and the scale bars are marked in each image.

To further explore the potential mechanism underlying PM_2.5_‐induced lung tumorigenesis, we conducted a circular RNA sequencing (circRNA‐seq) assay in control HBE and chronically PM_2.5_‐exposed cells (**Figure** **1**A). As shown in Figure 1B, 114 upregulated circRNAs and 123 downregulated circRNAs were detected after PM_2.5_ exposure, using a cut‐off fold change (FC) > 1.5 and a *p*‐value < 0.05. We then selected the top 5 circRNAs that were upregulated in PM_2.5_‐treated cells for quantitative real‐time reverse transcription polymerase chain reaction (qRT‐PCR) analysis. CircDNA2, which is located on chr10:70202673–70206170, exhibited the highest FC and was upregulated in a concentration‐dependent manner in PM_2.5_‐exposed HBE cells compared with the control group (Figure 1C; Figure S2, Supporting Information), and we therefore selected circDNA2 for further analysis.

**Figure 2:**
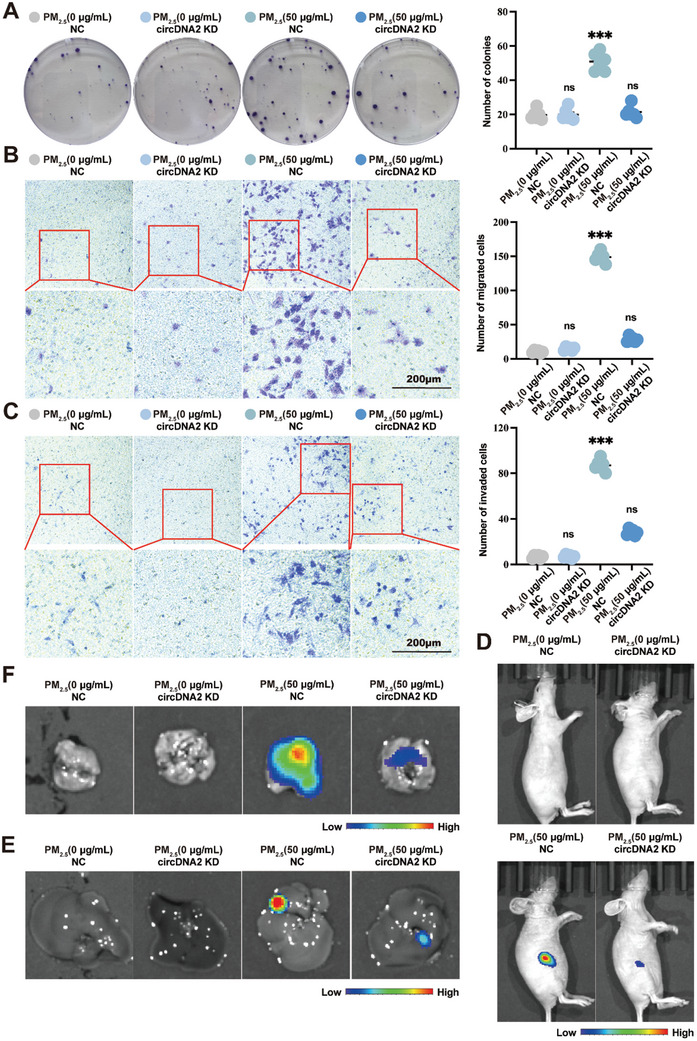
The function of circDNA2 in HBE cells subjected to long‐term PM_2.5_ exposure. A–C). Effects of circDNA2 KD on the colony formation A), migration ability B), and invasion ability C) of PM_2.5_‐transformed HBE cells in vitro (n = 6 per group, compared with the PM_2.5_ [0 µg mL^−1^] NC group, one‐way ANOVA, error bars represent SD, duplicate measurement per replicate). D–F). Effects of circDNA2 KD on PM_2.5_‐transformed HBE cells flank tumor burden D), and hepatic E), or lung F) metastasis in vivo (n = 6 per group, duplicate measurement per replicate). ns: no significant difference, ^***^ *p* < 0.001. Representative staining images are shown, and the scale bars are marked in each image.

CircDNA2 is a 476 nt circRNA derived from exons 8–10 of the *DNA2* gene located on chromosome 10. Sanger sequencing of PCR products confirmed the circular structure of circDNA2 (Figure 1D). Furthermore, circDNA2 could only be amplified using divergent primers from cDNA, but not from genomic DNA in HBE cells (Figure 1E), and showed strong resistance to RNase R (Figure 1F). To clarify the function of circDNA2, we examined its subcellular expression patterns. QRT‐PCR analysis of the cytosolic and nuclear fractions from HBE cells and RNA‐FISH analysis revealed that circDNA2 was enriched in both the cytoplasm and nucleus (Figure 1G,H). To confirm whether circDNA2 contributes to PM_2.5_‐triggered malignant transformation, we examined the effects of circDNA2 ablation on PM_2.5_‐transformed HBE cells. By infecting the PM_2.5_‐transformed HBE cells with the circDNA2 short hairpin RNA (shRNA) lentiviral GV112 vector, we established cell lines with stable knockdown (KD) of circDNA2 (Figure S3, Supporting Information). We found that circDNA2 KD reduced the growth of PM_2.5_‐transformed HBE cells (**Figure** **2**A) and their migratory and invasive abilities (Figure 2B,C). Consistently, circDNA2 KD also decreased the tumor growth and hepatic or lung metastatic ability of PM_2.5_‐transformed HBE cells (Figure 2D–F). Taken together, these results corroborated the involvement of circDNA2 in PM_2.5_‐induced malignant transformation as a pro‐carcinogenic factor.

### 2.3 CircDNA2 Promotes PM_2.5_‐Induced Malignant Transformation by Inhibiting GADD45A Expression

**Figure 3:**
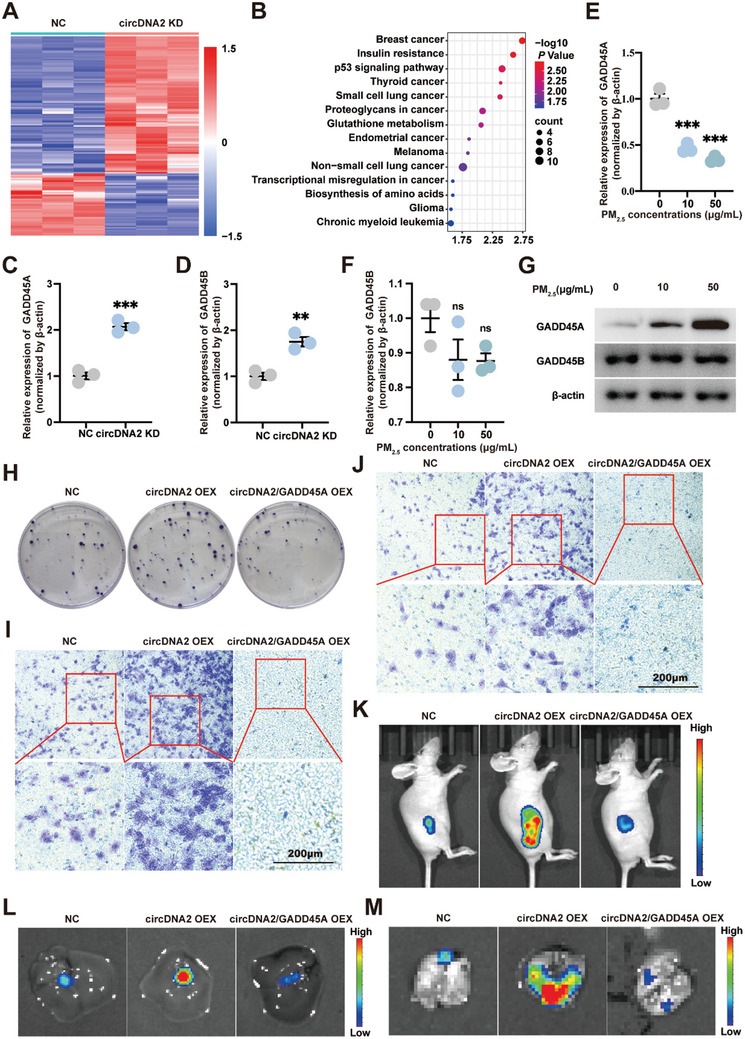
CircDNA2 promotes PM_2.5_‐induced malignant transformation by inhibiting GADD45A expression. A). Three pairs of HBE cells in vehicle control (NC) and circDNA2 KD group were subjected to RNA‐seq assay. Heatmaps revealed differentially expressed mRNAs. B). KEGG pathway analysis of RNA‐seq. C–D). Expression levels of GADD45A C) and GADD45B D) of HBE cells in NC and circDNA2 KD group (n = 3 per group, compared with NC, two‐tailed t‐test, error bars represent SD). E–F). GADD45A E) and GADD45B F) mRNA levels in PM_2.5_‐transformed HBE cells (n = 3 per group, compared with the group with 0 µg mL^−1^ exposure concentration of PM_2.5_, one‐way ANOVA, error bars represent SD, duplicate measurement per replicate). G). GADD45A and GADD45B protein levels in PM_2.5_‐transformed HBE cells. H–J). Effects of circDNA2 and/or GADD45A OEX on the colony formation H), migration ability I), and invasion ability J) of PM_2.5_‐transformed HBE cells in vitro (n = 6 per group, duplicate measurement per replicate). K–M). Effects of circDNA2 and/or GADD45A OEX on PM_2.5_‐transformed HBE cells flank tumor burden K), and hepatic L), or lung M) metastasis in vivo (n = 6 per group, duplicate measurement per replicate). ns: no significant difference, ^**^ *p* < 0.01, ^***^ *p* < 0.001. The requirement for the GADD45A and GADD45B expression PM_2.5_‐transformed HBE cells was confirmed by at least one additional independent experiment. Representative staining images are shown. Representative staining images are shown, and the scale bars are marked in each image.

Next, to clarify the molecular mechanism by which circDNA2 regulates PM_2.5_‐triggered malignant transformation, circDNA2 target genes were screened via RNA sequencing (RNA‐seq) analysis of HBE cells in the vehicle control (NC) and circDNA2 KD groups (**Figure** **3**A). Kyoto Encyclopedia of Genes and Genomes (KEGG) enrichment analysis showed that the differentially expressed genes were enriched in the small cell lung cancer and non‐small cell lung cancer pathways (Figure 3B), which included two shared genes, growth arrest and DNA damage 45 alpha (GADD45A) and beta (GADD45B). Subsequent qRT‐PCR results also confirmed that both genes were potential targets of circDNA2 (Figure 3C,D). However, when HBE cells were exposed to PM_2.5_, only the expression of GADD45A was downregulated in a dose‐dependent manner, whereas no corresponding change in GADD45B expression was observed (Figure 3E–G). Therefore, GADD45A was selected as the target for follow‐up experiments.

First, by infecting the PM_2.5_‐transformed HBE cells (exposed to PM_2.5_ [50 µg mL^−1^] for 30 generations) with circDNA2 overexpression (OEX) lentivirus and GADD45A OEX lentivirus, we established the cell lines of circDNA2 OEX and/or GADD45A OEX (Figure S4A, Supporting Information). Subsequent experiments were performed using the newly established cell lines. As expected, when circDNA2 was overexpressed, the proliferation, migration, and invasion abilities of malignantly transformed HBE cells induced by PM_2.5_ were significantly enhanced in vitro, as well as tumor growth and metastasis in vivo. However, these effects caused by circDNA2 OEX were attenuated when GADD45A was overexpressed (Figure 3H–M; Figure S4B, Supporting Information), implying that GADD45A acts downstream of circDNA2. Therefore, we determined that circDNA2 was involved in the PM_2.5_‐induced malignant transformation of HBE cells by regulating the downstream molecule GADD45A.

### 2.4 CircDNA2 Inhibits GADD45A Expression by Interacting with the LC Domain of the m^6^A Reader Protein YTHDF2

**Figure 4:**
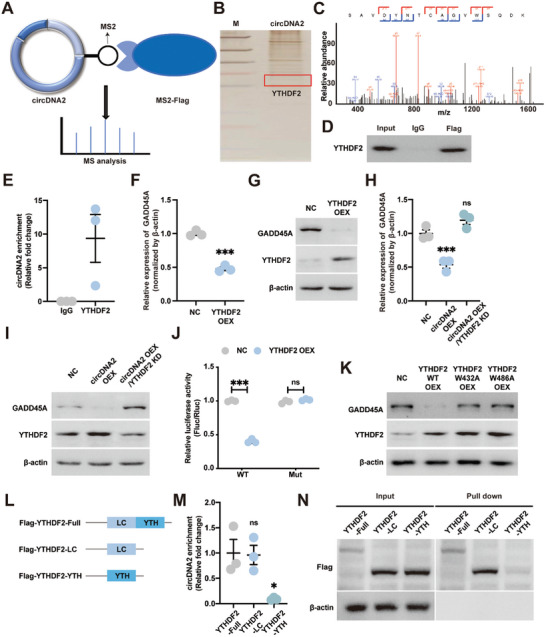
CircDNA2 inhibits GADD45A expression by interacting with the LC domain of the m^6^A reader protein YTHDF2. A). Schematic diagram of the RNA pull‐down assay performed using the MS2‐tagging system. B). Silver‐stained circDNA2 binding proteins derived from RNA pull‐down. C). The typical YTHDF2 peptide identified among circDNA2‐enriched proteins. D). Confirmation of the binding of circDNA2 and YTHDF2 using the MS2‐tagging system. E). Confirmation of the binding of circDNA2 and YTHDF2 using RIP assay. F–G). Effects of YTHDF2 OEX on GADD45A mRNA F) and protein G) levels in PM_2.5_‐transformed HBE cells (n = 3 per group, compared with NC, two‐tailed *t*‐test, error bars represent SD, duplicate measurement per replicate). H–I). Effects of circDNA2 OEX and/or YTHDF2 KD on GADD45A mRNA H) and protein I) levels in PM_2.5_‐transformed HBE cells (n = 3 per group, compared with NC, one‐way ANOVA, error bars represent SD, duplicate measurement per replicate). J). Relative activity of WT or mutant (Mut) GADD45A mRNA firefly luciferase reporters in vehicle control (NC) and YTHDF2 OEX PM_2.5_‐transformed HBE cells (n = 3 per group, compared with NC within each group, two‐tailed *t*‐test, error bars represent SD, duplicate measurement per replicate). K). Effects of m^6^A recognition domain mutant YTHDF2 OEX on GADD45A and YTHDF2 protein levels in PM_2.5_‐transformed HBE cells (n = 3 per group, duplicate measurement per replicate). L). Structure of FLAG‐tagged wild‐type or truncated YTHDF2. M). RIP‐qPCR detecting the binding of FLAG‐tagged YTHDF2 to circDNA2 in PM_2.5_‐transformed HBE cells (n = 3 per group, compared with the wild‐type, one‐way ANOVA, error bars represent SD, duplicate measurement per replicate). N). RNA pull‐down was used to detect the binding of FLAG‐tagged YTHDF2 to circDNA2 in PM_2.5_‐transformed HBE cells. ns: no significant difference, ^*^ *p* < 0.05, ^***^ *p* < 0.001. All experiments were performed with PM_2.5_ (50 µg mL^−1^)‐transformed HBE cells. The requirement for the YTHDF2 or Flag taged YTHDF2 level in the pull down assay was confirmed by at least one additional independent experiment.

Given that GADD45A mRNA was negatively regulated by circDNA2 (Figure 3C), we speculate that circDNA2 inhibited GADD45A mRNA expression by binding to proteins rather than through a traditional competing endogenous RNAs (ceRNA) mechanism. To this end, we screened the proteins that could bind to circDNA2 using an MS2‐tagged pull‐down assay. Briefly, circDNA2‐MS2 and flag‐MS2 vectors were co‐transfected into HBE cells. Potential circDNA2‐binding proteins were detected using FLAG antibody‐coated magnetic beads, silver staining, and mass spectrometry (MS) analysis (**Figure** **4**A). Among the proteins that were found to potentially bind to circDNA2, we identified the N6‐methyladenosine (m^6^A) reader protein YTH N6‐Methyladenosine RNA Binding Protein F2 (YTHDF2), which regulates mRNA stability in an m^6^A‐dependent manner (Figure 4B,C; Figure S5, Supporting Information). Therefore, YTHDF2 was selected as the potential circDNA2 target for further analysis. The MS2‐tagged pull‐down assay and YTHDF2 RNA‐binding protein immunoprecipitation‐quantitative real‐time reverse transcription polymerase chain reaction (RIP‐qPCR) assays further confirmed that circDNA2 interacted with YTHDF2 (Figure 4D,E).

Both qRT‐PCR and western blotting (WB) confirmed that YTHDF2 OEX inhibited GADD45A expression (Figure 4F,G) and that the inhibitory effect of circDNA2 OEX on GADD45A was attenuated by YTHDF2 KD (Figure 4H,I). As expected, YTHDF2 OEX decreased the luciferase activity of wild‐type (WT) GADD45A reporter, whereas this decrease was abolished by mutations in the m^6^A consensus site of GADD45A mRNA (Figure 4J). The YTHDF2 m^6^A recognition mutants, W432A and W486A, abolished the effect of YTHDF2 on GADD45A protein levels (Figure 4K). To further clarify the regions of interaction between circDNA2 and YTHDF2, we performed a Flag‐RIP assay using HBE cells overexpressing the truncated YTHDF2. Results showed that the LC domain of YTHDF2 was essential for its interaction with circDNA2 (Figure 4L,M). A pull‐down assay confirmed the interaction between the LC domain of YTHDF2 and circDNA2 (Figure 4N). In summary, our data demonstrated that circDNA2 regulates GADD45A expression by binding to the YTHDF2 LC domain.

### 2.5 CircDNA2 Promotes YTHDF2 LLPS by Binding to the YTHDF2 LC Domain

**Figure 5:**
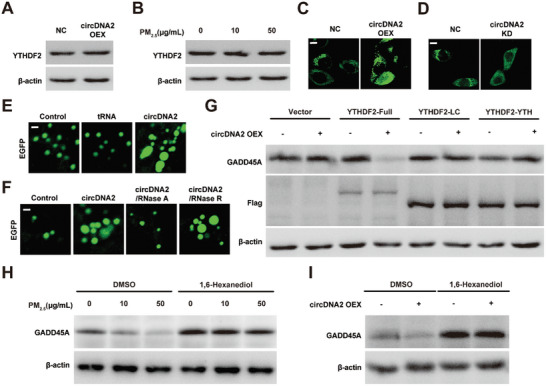
CircDNA2 promotes YTHDF2 LLPS by binding to the YTHDF2 LC domain. A–B). YTHDF2 protein levels in circDNA2 OEX A) or PM_2.5_‐transformed B) HBE cells. C). Representative fluorescent images of LLPS in control and circDNA2 OEX HBE cells (scale bar, 10 µm). D). Representative fluorescent images of LLPS in control and circDNA2 KD cells (scale bar, 10 µm). E). In vitro LLPS of purified YTHDF2‐EGFP with or without different types of RNA (scale bar, 5 µm). F). In vitro LLPS of purified YTHDF2‐EGFP with circDNA2 with or without different types of RNase (scale bar, 5 µm). G). Effects of circDNA2 on GADD45A protein expression reconstituted with YTHDF2‐Full, YTHDF2‐LC, or YTHDF2‐YTH. H–I). Effects of 1,6‐Hexanediol on GADD45A protein expression in PM_2.5_ (50 µg mL^−1^)‐transformed H) and circDNA2 OEX I) HBE cells. The requirement for the YTHDF2 expression in circDNA2 OEX or PM_2.5_‐transformed HBE cells, and GADD45A expression in circDNA2 OEX and YTHDF2 KO HBE cells, as well as GADD45A expression in PM_2.5_‐transformed or circDNA2 OEX(PM_2.5_‐transformed) HBE cells treated with 1,6‐Hexanediol, was confirmed by at least one additional independent experiment. Representative staining images are shown.

To explore the potential mechanism by which circDNA2/YTHDF2 regulates GADD45A expression, we initially detected whether circDNA2 and PM_2.5_ exposure were able to regulate YTHDF2 expression. Our results showed that YTHDF2 protein expression was not significantly affected by circDNA2 OEX and chronic low‐level PM_2.5_ exposure (**Figure** **5**A,B). Given the absence of changes in protein expression, we speculated that an alternative mechanism of action, such as phase separation, might be involved. In particular, the key role of the YTHDF2 LC drew our attention. The LC domain of YTHDF2 has the potential to form liquid‐liquid phase separation (LLPS), and circRNAs are associated with LLPS‐related events.^[^ ^11^, ^12^ ^]^ As we had already confirmed the specific binding between circDNA2 and the YTHDF2 LC domain (Figure 4), we hypothesized that circDNA2 promoted YTHDF2 LLPS by binding to the YTHDF2 LC domain. We found that circDNA2 OEX significantly increased YTHDF2 puncta formation, whereas circDNA2 KD inhibited YTHDF2 puncta formation in HBE cells in vivo (Figure 5C,D). Moreover, circDNA2 promoted YTHDF2 droplet formation in vitro (Figure 5E). Furthermore, this enhancement was attenuated by RNase A, but not by RNase R, suggesting that YTHDF2 droplet formation was mediated by circDNA2 (Figure 5F). We then explored whether YTHDF2 LLPS was required for circDNA2‐induced phenotypes. We first constructed and overexpressed YTHDF2 full‐Flag, YTHDF2 LC‐Flag, and YTHDF2 YTH‐Flag vectors in HBE cells. As depicted in Figure 5G, only when YTHDF2 retained its ability to induce LLPS and recognize m^6^A was circDNA2 able to suppress GADD45A expression. Meanwhile, the addition of a phase separation inhibitor (1,6‐Hexanediol) hindered the regulation of GADD45A expression induced by PM_2.5_ or circDNA2 (Figure 5H,I). In conclusion, these results confirmed that circDNA2 promotes YTHDF2 LLPS by binding to the YTHDF2 LC domain.

### 2.6 CircDNA2 and GADD45A are Potential Prognostic Biomarkers for Lung Cancer

**Figure 6:**
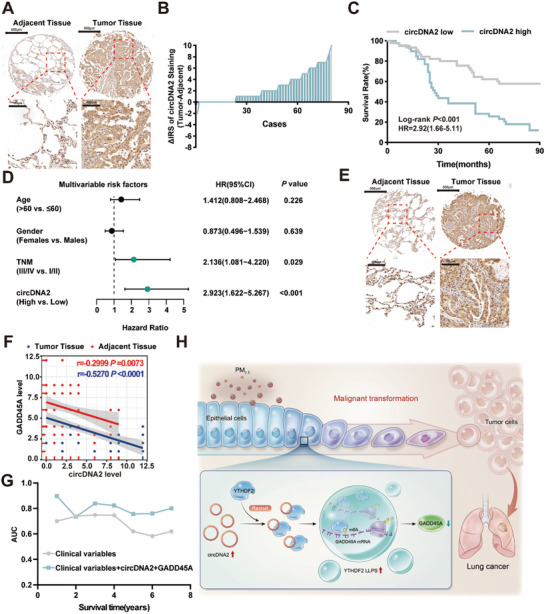
CircDNA2 and GADD45A are potential prognostic biomarkers for lung cancer. A–B). The levels of circDNA2 in TMAs were evaluated by ISH, and the differences in staining scores between lung cancer lesions and adjacent tissues are shown. C). KM curves depicting the overall survival of patients with lung cancer. D). Multivariate Cox regression analysis of patients with lung cancer. E). The levels of GADD45A in TMAs were evaluated by immunohistochemistry (IHC). F) Correlation between GADD45A and circDNA2 expression levels in TMA (n = 77 for tumor tissues, and n = 79 for adjacent tissues, Pearson correlation test). G). ROC analysis of patients with lung cancer. H). The systemic molecular pathway of circDNA2‐mediated tumorigenesis. Representative staining images are shown, and the scale bars are marked in each image.

Given the important role of circDNA2 in PM_2.5_‐induced malignant transformation of HBE cells, we further explored the potential role of circDNA2 in lung cancer. First, we detected circDNA2 expression in 90 pairs of lung cancer and adjacent non‐tumor tissues through an in situ hybridization (ISH) staining analysis of tissue microarrays (TMAs). We observed significantly increased circDNA2 expression in lung cancer tissues (**Figure** **6**A,B) and found that elevated tumor circDNA2 levels were significantly correlated with decreased overall survival time in patients with lung cancer (Figure 6C). Multivariate Cox regression analysis indicated that low circDNA2 expression was an independent prognostic factor for lung cancer (Figure 6D). Similarly, we confirmed low expression of GADD45A in lung cancer tissues, which was also an independent poor prognostic factor for lung cancer (Figure 6E; Figure S6, Supporting Information). Moreover, GADD45A levels negatively correlated with circDNA2 expression (Figure 6F). To further evaluate the prognostic value of circDNA2 and GADD45A expressions, we performed a time‐dependent receiver operating characteristic curve (ROC) analysis of the censored data, which showed that the prognostic model, including circDNA2 and GADD45A, outperformed clinical indicators as predictors of patient survival (Figure 6G).

## 3 Discussion

By constructing a chronic PM_2.5_ exposure model, we first confirmed that PM_2.5_ exposure induces the malignant transformation of HBE cells. Bioinformatics analysis combined with molecular biology experiments revealed that circDNA2, which was abnormally upregulated after exposure to PM_2.5_, promoted the degradation of GADD45A mRNA in a YTHDF2‐mediated m^6^A‐dependent manner and that circDNA2 specifically bound to the LC domain of YTHDF2 to promote YTHDF2 LLPS. Finally, we evaluated the clinical value of circDNA2 and GADD45A as potential prognostic biomarkers for lung cancer using the clinical information from patients with lung cancer (Figure 6H).

Despite numerous epidemiological studies confirming that PM_2.5_ is a risk factor for lung cancer, the molecular mechanism underlying PM_2.5_‐induced lung cancer remains largely unknown.^[^ ^6^ ^]^ Recently, circRNAs have emerged as a popular topic in the field of epigenetics because of their specificity and stability of expression, and they are regarded as particularly promising diagnostic markers and therapeutic targets.^[^ ^13^ ^]^ Although circRNAs are involved in the development and progression of various respiratory diseases, there has been little focus on the role of circRNAs as intermediaries to study their mechanistic functions in PM_2.5_‐induced lung cancer.^[^ ^8^, ^14^ ^]^ Therefore, in this study, three pairs of HBE cells exposed to different concentrations of PM_2.5_ were subjected to a circRNA‐seq assay, and circDNA2 was screened out with significantly elevated expression after PM_2.5_ exposure as the core concern gene through bioinformatics analysis and molecular biology experiments. In addition, the relationship among circDNA2, PM_2.5_ exposure, and lung cancer has not been reported. Therefore, we focused on elucidating the regulatory mechanisms linking these factors. As circRNAs with different localization patterns function in different ways, initial analyses of a given circRNA hinge on detecting its localization.^[^ ^15^ ^]^ Nucleocytoplasmic separation and RNA‐FISH analyses showed that circDNA2 was enriched in both the cytoplasm and nucleus, in contrast to the traditional circRNAs expressed in the cytoplasm, which mostly function as ceRNAs.^[^ ^14^, ^16^ ^]^ Therefore, in this study, we focused on the potential protein‐binding functions of circDNA2. Transcriptomic sequencing, bioinformatics prediction, and molecular biology experiments ultimately confirmed that GADD45A was a downstream molecule of circDNA2.

The m^6^A RNA modification, a typical epigenetic alteration that contributes to gene expression regulation, is involved in environmental pollutant‐induced tumorigenesis.^[^ ^17^ ^]^ For example, the RNA m^6^A demethylase ALKBH5 upregulates ZKSCAN3 expression, which in turn activates VEGFA transcription, thus participating in MNNG‐induced gastric cancer progression.^[^ ^18^ ^]^ The m^6^A writer METTL3 upregulates AKT1 mRNA levels by increasing YTHDF1‐mediated AKT1 mRNA stability and induces AKT1 phosphorylation at the S473 and T308 residues by regulating PHLPP2 and PDK1 expression in arsenic‐induced carcinogenesis.^[^ ^19^ ^]^ Consistent with these previous studies, our results confirmed the pivotal role of m^6^A RNA modification in PM_2.5_‐induced lung cancer, specifically, circDNA2 inhibits GADD45A expression via interaction with the LC domain of the m^6^A reader protein YTHDF2.

LLPS, a reversible physiological process driven by the cumulative interactions between proteins or nucleic acids, plays a pivotal role in various biological activities, such as gene transcription, genome organization, epigenetic modification, and signal transduction.^[^ ^20^ ^]^ LLPS can concentrate molecules in condensates, thereby enhancing reactions, signaling, and cytoskeletal nucleation. Condensates can accelerate biochemical reactions by increasing the local concentrations of enzymes or protein complexes.^[^ ^21^ ^]^ For example, ATXN2 phase separation recruits ribosomes and selective mRNAs to facilitate translational activation during the circadian cycle.^[^ ^22^ ^]^ KAT6A LLPS enhances the interaction between KAT6A and PARP1 in ovarian cancer (KAT6A condensation impairs PARP1 trapping by PARP inhibitors in ovarian cancer). Therefore, we speculated that YTHDF2 LLPS might concentrate molecules, including GADD45A mRNA, in condensates, ultimately accelerating GADD45A mRNA degradation. Additionally, LLPS formation is highly responsive to physicochemical cues. Even minor alterations in RNA modifications, energy input, and physicochemical conditions (such as pH, ionic salt strength, and temperature) can regulate LLPS.^[^ ^23^ ^]^ CircRNAs have been shown to regulate cancer progression by participating in LLPS events.^[^ ^11^, ^12^ ^]^ Moreover, YTHDF2 contains a low‐complexity LC domain at its N‐terminus, which has the potential to generate LLPS, and the m^6^A recognition domain at its C‐terminus (YTH domain) is of great significance for the phase separation phenomenon mediated by YTHDF2 itself.^[^ ^24^ ^]^ As we confirmed the specific binding between circDNA2 and YTHDF2 LC domains, we sought to further explore whether circDNA2 was able to promote YTHDF2 LLPS. We successfully demonstrated that circDNA2 promotes YTHDF2 LLPS by binding to the YTHDF2 LC domain during the malignant transformation of lung epithelial cells. To the best of our knowledge, this is the first evidence linking LLPS to particulate pollutant‐induced tumorigenesis.

GADD45A, a tumor suppressor gene in several cancers, regulates cell cycle arrest, cell survival, and apoptosis in response to physiological and environmental stresses. GADD45A induces G2/S‐phase cell cycle arrest by interacting with cyclin B1, cyclin D3, and CDC2,^[^ ^25^ ^]^ and triggers apoptosis by activating the JNK/SAPK signaling pathway.^[^ ^26^ ^]^ In lung cancer, GADD45A KD suppresses G2/M cell cycle arrest and apoptosis via the JNK/P38 pathway.^[^ ^27^ ^]^ In our study, we found that during the PM_2.5_‐induced malignant transformation of lung epithelial cells, GADD45 was downregulated by circDNA2 in a YTHDF2‐mediated, m^6^A‐dependent manner. Moreover, GADD45A attenuated PM_2.5_ and circDNA2‐induced tumorigenesis in the transformed HBE cells. Taken together, these results corroborate the ability of GADD45A to exert tumor suppressor functions in PM_2.5_‐induced malignant transformation of lung epithelial cells, and m^6^A modification‐mediated post‐transcriptional regulation may serve as a key mechanism for the downregulation of GADD45A in lung cancer.

Although this study elaborated on the important role of circDNA2 in PM_2.5_‐induced lung cancer carcinogenesis and development, some areas require improvement in subsequent studies. For example, the absence of animal models of lung cancer induced by PM_2.5_ is a limitation of this study. We look forward to further explorations using animal models of lung cancer in our subsequent studies. Furthermore, owing to the potential interaction between fetal bovine serum (FBS) and PM_2.5_ which affects the toxicity of PM_2.5_,^[^ ^28^ ^]^ further studies are required to investigate this matter. Moreover, although PM_2.5_ Standard Reference Material (NIST 1648a) was used in this study, it still cannot fully represent the PM_2.5_ present in the real world. Studies involving actual PM_2.5_ samples should be conducted to validate our findings.

## 4 Conclusion

In summary, we revealed the mechanism by which circDNA2‐educated YTHDF2 phase separation promotes PM_2.5_‐induced malignant transformation in HBE cells by blunting GADD45A expression, providing definitive evidence that LLPS is involved in the induction of tumorigenesis by particulate pollutants. Moreover, we evaluated the clinical value of circDNA2 and GADD45A as potential prognostic biomarkers of lung cancer. This study provides new insights that guide the exploration of the mechanistic roles of circRNAs in PM_2.5_‐induced lung cancer.

## 5 Experimental Section

Complete details of all reagents and procedures used in this study are provided in the SI Appendix, SI Materials and Methods.

### Chemicals

PM_2.5_ (Standard Reference Material 1648a) was purchased from the National Institute of Standards & Technology (NIST; USA). Phosphate‐buffered saline (PBS; Gibco, NY, USA) was used to store PM_2.5_ at a concentration of 100 mg mL^−1^. The stock solutions were ultrasonicated for 30 min before appropriate dilution.

### Cell Lines

HBE (HBE135‐E6E7) cells were sourced from the American Type Culture Collection (ATCC; USA). The cells were cultured in complete Dulbecco's modified Eagle's medium (DMEM; Gibco) supplemented with 10% (v/v) FBS (REF: 10270–106, Gibco), 100 U mL^−1^ penicillin (Gibco), and 100 µg mL^−1^ streptomycin (Gibco) under 5% CO_2_ at 37 °C. Regular mycoplasma contamination tests and cell line authentication were conducted to validate in vitro experiments.

### Long‐Term PM_2.5_ Exposure Study

As reported in the prior study,^[^ ^29^ ^]^ the HBE cells were cultured for 48–72 h in a medium containing PM_2.5_ concentrations of 0, 10, or 50 µg mL^−1^ (the concentration was determined based on the results of the previous studies).^[^ ^30^ ^]^ Subsequently, the cells were passaged to a medium without PM_2.5_ and subcultured for two generations. This process was repeated 10 times, resulting in 30 passages over 3 months.

### Establishment of Stable Cell Lines

Lentiviral vector construction and establishment of stable cell clones firefly_luciferase OEX lentiviral GV542 vector (Ubc‐MCS‐SV40‐firefly_luciferase‐IRES‐neomycin), YTHDF2 (W432A/W486A) and GADD45A OEX lentiviral GV341 vector (Ubc‐MCS‐3FLAG‐SV40‐puromycin), YTHDF2 and circDNA2 shRNA lentiviral GV112 vector (hU6‐MCS‐CMV‐Puromycin), circDNA2 OEX lentiviral GV689 vector (CMV‐circDNA2‐EF1a‐Zsgreen1‐T2A‐puromycin), and control lentiviral vectors were synthesized by Shanghai Genechem Co. Ltd. (Shanghai, China). The HBE cells were infected at a multiplicity of infection of 10, according to the manufacturer's protocol. Then, the cells were selected with 500 µg mL^−1^ G418 (Beyotime Biotechnology, Shanghai, China) or 2 µg mL^−1^ puromycin (Sigma‐Aldrich, Louis, USA) to generate stable cell lines. The expression levels of the target genes in infected HBE cells were confirmed by qRT‐PCR or WB assay.

### Animal Experiments

All experiments were performed in accordance with the guidelines of the Committee on Animal Use and Care of the Capital Medical University (approval number: AEEI‐2022‐010). Six‐week‐old female BALB/c Nude Mice were purchased from the Vital River Laboratory Animal Technology Co., Ltd (Beijing, China) and kept in a 12‐h/12‐h light/dark cycle environment with an indoor temperature of 24 °C ± 2 °C and relative humidity of 50% ± 5%. Three murine models were established in this study.

The first type of murine model was a xenograft model used to detect the malignancy of cells injected into mice (Figures 2D and 3K; Figure S1E, Supporting Information). In this model, the mice were injected subcutaneously into their left dorsal flanks with 0.1 mL mouse^−1^ of a cell suspension containing 5 × 10^6^ cells on day 1. The malignancy of the cells (tumor growth) was examined based on the size and numerical value of the fluorescent area in the image using an In Vivo Imaging System (IVIS) Spectrum Imaging System (Xenogen, CA, USA) on day 14.

The second type of murine model was the hepatic metastasis model, which was used to investigate the metastatic ability of cells injected into mice (Figures 2E and 3L; Figure S1F, Supporting Information). In this model, the mice were intrasplenically injected with 0.1 mL mouse^−1^ of a cell suspension containing 1 × 10^6^ cells on day 1. The fluorescence intensity in the liver was also assessed using IVIS on day 14 to evaluate the metastatic ability of the cells.

Finally, the third murine model was the lung metastasis model, which was constructed to re‐evaluate the metastatic ability of cells injected into mice (Figures 2F and 3M; Figure S1G, Supporting Information). Briefly, a total of 0.1 mL mouse^−1^ of a cell suspension containing 1 × 10^6^ cells was injected into the mice via the tail vein. The pulmonary metastatic potential of the cells was assayed 14 days after injection by IVIS.

### CircRNA‐seq and Data Analysis

For the circRNA‐seq assay, total RNA was isolated from cells using the Hipure Total RNA Mini Kit (Magen, Guangzhou, China), according to the manufacturer's protocols. ≈2 µg of total RNA was used to prepare the sequencing library with a KAPA RNA HyperPrep Kit with RiboErase for Illumina^@^ (Kapa Biosystems, Inc., MA, USA), following the manufacturer's recommendations. After a quality check using an Agilent Bioanalyzer 2100 system, paired‐end (PE150) sequencing was performed for all samples. Each group was sequenced in triplicates. For circRNA‐seq data analysis, reads were mapped to the genome using STAR, and DCC was used to identify and estimate circRNA expression. Differentially expressed genes (DEGs) were identified using the edgeR program. FC > 1.5 and *p* < 0.05 were considered statistically significant for differentially expressed circRNAs. R was used to generate the figure. The high‐throughput data were uploaded to the Gene Expression Omnibus (GEO) database (circRNA‐seq: GSE283083).

### RIP‐qPCR Assay

RIP assays were conducted using a Magna RIP RNA‐Binding Protein Immunoprecipitation Kit (Cat: 17–700, Merck KGaA), according to the manufacturer's instructions. Briefly, magnetic beads coated with antibodies against mouse immunoglobulin G (Cat: SAB5600195, Merck KGaA), GADD45A (Cat: Ag21836, Proteintech, Hubei, China), or FLAG (Cat: F3165, Merck KGaA) were incubated overnight at 4 °C with pre‐frozen cell extracts. The RNA–protein complexes were then collected, washed six times, and subjected to proteinase K (Thermo Fisher Scientific, CA, USA) digestion and RNA extraction using the TRIzol reagent (Thermo Fisher Scientific). The extent of the protein–RNA interaction was assessed using qRT‐PCR and normalized against the input levels.

### RNA‐Seq and Data Analysis

Total RNA was isolated from HBE cells using the TRIzol reagent (Thermo Fisher Scientific). ≈3 µg of total RNA was used to prepare the sequencing library with a NEBNext UltraTM RNA Library Prep Kit for Illumina (New England Biolabs, Inc., MA, USA), following the manufacturer's recommendations. After a quality check using the Agilent Bioanalyzer 2100 system, the libraries were sequenced using the Illumina HiSeq platform. Each group was sequenced in triplicates.

For the RNA‐seq data analysis, reads containing adapter, poly‐N, and low‐quality reads were removed to obtain clean reads. All reads were aligned to the genome using HiSat2 v2.0.5. The Counts v1.5.0‐p3 feature was used to count the read numbers mapped to each gene and the FPKM value for each gene was calculated to estimate gene expression. Ballgown (v2.10.0) was used to determine DEGs between conditions with an absolute FC > 1.5 and with *p* < 0.05. KEGG enrichment analysis was performed using https://david.ncifcrf.gov/. Heatmaps were generated using the pheatmap (v1.0.12) in R (v4.1.0). The high‐throughput data were uploaded to the GEO database (RNA‐seq: GSE283081).

### Cellular Phase Separation Assay

HBE cells expressing YTHDF2‐EGFP were seeded onto confocal dishes. After adherence, the cells were infected with circDNA2 OEX or KD vectors and finally analyzed by confocal microscopy.

### In vitro Phase Separation Assay

In vitro phase separation assays were conducted in a storage buffer with varying concentrations of purified proteins, and PEG8000 (Beyotime Biotechnology, Shanghai, China) was added at a final concentration of 10% (w/v). The mixtures were imaged using a confocal microscope.

### Statistical Analysis

Data were presented as means ± standard deviations (SD). The 2^−ΔΔCt^ method was used to analyze the results of qRT‐PCR analyses. Continuous data were compared among multiple groups using one‐way analysis of variances (ANOVAs) or two‐way ANOVAs, or between two groups using *t*‐tests, as indicated in the figure legends. All statistical analyses were performed using SAS software (v9.4). Differences were considered statistically significant at *p* < 0.05. Histograms were constructed using GraphPad Prism (v9.0).

### Ethics Approval

The study was approved by the Ethics Committee of the Capital Medical University. All animal experiments were performed in accordance with the guidelines of the Committee on Animal Use and Care of Capital Medical University (approval number: AEEI‐2022‐010).

## Conflict of Interest

The authors declare no conflict of interest.

## Supporting information



Supporting Information

## Data Availability

The data that support the findings of this study are available from the corresponding author upon reasonable request.
