# Supplementary material for: CircDNA2‐Educated YTHDF2 Phase Separation Promotes PM2.5‐Induced Malignant Transformation Through the Blunting of GADD45A Expression
Source: Adv Sci (Weinh). 2025 Jan 17;12(14):2410532. doi: 10.1002/advs.202410532 (PMC11984909; doi:10.1002/advs.202410532)
Supplement: Supplementary file 1 — Supporting Information [file ADVS-12-2410532-s001.docx]

**CircDNA2-educated YTHDF2 Phase Separation Promotes PM_2.5_-induced Malignant Transformation through the Blunting of GADD45A Expression**

Jie Xu^1^, Zhi Ling^2^, Lijia Yin^2^, Duo Xu^2^, Shenshen Wu^2*^, Rui Chen^2, 3, 4, 5*^

^1^ Yunnan Provincial Key Laboratory of Public Health and Biosafety & School of Public Health, Kunming Medical University, Kunming 650500, P. R. China

^2^ School of Public Health, Capital Medical University, Beijing 100069, P. R. China

^3^ Laboratory for Environmental Health and Allergic Nasal Diseases, Laboratory for Clinical Medicine, Capital Medical University, Beijing 100069, P. R. China

^4^ Beijing Laboratory of Allergic Diseases, Capital Medical University, Beijing 100069, P. R. China

^5^ Department of Occupational and Environmental Health, Fourth Military Medical University, Ministry of Education Key Lab of Hazard Assessment and Control in Special Operational Environment, Xi’an 710032, P. R. China

**^*^Corresponding authors at:** School of Public Health, Capital Medical University, Beijing 100069, China.

***E-mail addresses*:** sswu@ccmu.edu.cn (S. Wu), ruichen@ccmu.edu.cn (R. Chen).

^*^ These authors contributed equally to this work.

**Authors’ information:** Jie Xu, xujie@kmmu.edu.cn; Zhi Ling, 15633400601@163.com; Lijia Yin, 15735012664@163.com; Duo Xu, 17852322930@163.com; Shenshen Wu, sswu@ccmu.edu.cn; Rui Chen, ruichen@ccmu.edu.cn.

**1. SI Materials and Methods**

**1.1 Establishment of stable cell lines (supplementary)**

In detail, a total of ten stable cell lines were constructed in this article, specifically as follows:

(1) Particulate matter 2.5 (PM_2.5_; 0 μg/mL) + NC (Figure 2 and Figure S3, Supporting Information) cells: the PM_2.5_ (0 μg/mL)-transformed human bronchial epithelial (HBE) cells were infected with control lentivirus.

(2) PM_2.5_ (0 μg/mL) + circDNA2 knockdown (KD; Figure 2 and Figure S3, Supporting Information) cells: the PM_2.5_ (0 μg/mL)-transformed HBE cells were infected with circDNA2 short hairpin RNA (shRNA) lentivirus.

(3) PM_2.5_ (50 μg/mL) + NC (Figure 2 and Figure S3, Supporting Information) (marked as “NC” in Figure 3-5) cells: the PM_2.5_ (50 μg/mL)-transformed HBE cells were infected with control lentivirus.

(4) PM_2.5_ (50 μg/mL) + circDNA2 KD (Figure 2 and Figure S3, Supporting Information) (marked as “circDNA2 KD” in Figure 3, 5) cells: the PM_2.5_ (50 μg/mL)-transformed HBE cells were infected with circDNA2 shRNA lentivirus.

(5) PM_2.5_ (50 μg/mL) + circDNA2 overexpression (OEX; marked as “circDNA2 OEX” in Figure 3-5 and Figure S4, Supporting Information) cells: the PM_2.5_ (50 μg/mL)-transformed HBE cells were infected with circDNA2 OEX lentivirus.

(6) PM_2.5_ (50 μg/mL) + circDNA2/GADD45A OEX (marked as “circDNA2/GADD45A OEX” in Figure 3 and Figure S4, Supporting Information) cells: the PM_2.5_ (50 μg/mL)-transformed HBE cells were co-infected with circDNA2 OEX lentivirus and GADD45A OEX lentivirus.

(7) PM_2.5_ (50 μg/mL) + YTHDF2 OEX (marked as “YTHDF2 (WT) OEX” in Figure 4) cells: the PM_2.5_ (50 μg/mL)-transformed HBE cells were infected with YTHDF2 OEX lentivirus.

(8) PM_2.5_ (50 μg/mL) + circDNA2 OEX/YTHDF2 KD (marked as “circDNA2 OEX/YTHDF2 KD” in Figure 4) cells: the PM_2.5_ (50 μg/mL)-transformed HBE cells were co-infected with circDNA2 OEX lentivirus and YTHDF2 shRNA lentivirus.

(9) PM_2.5_ (50 μg/mL) + YTHDF2 W432A OEX (marked as “YTHDF2 W432A OEX” in Figure 4) cells: the PM_2.5_ (50 μg/mL)-transformed HBE cells were infected with YTHDF2 W432A OEX lentivirus.

(10) PM_2.5_ (50 μg/mL) + YTHDF2 W486A OEX (marked as “YTHDF2 W486A OEX” in Figure 4) cells: PM_2.5_ (50 μg/mL)-transformed HBE cells were infected with YTHDF2 W486A OEX lentivirus.

**1.2 Analysis of cell colony formation, invasion, and migration ability**

Briefly, for colony formation assays, 1000 HBE cells were seeded in 10-cm culture dishes (Corning Incorporated, NY, USA) and allowed to grow in complete culture medium for 10 days. For cell invasion assays, 1 × 10^5^ cells (in 200 μL Dulbecco’s modified Eagle’s medium [DMEM; Gibco, NY, USA] with 0.5% foetal bovine serum [FBS; Gibco]) were seeded into the upper chamber of the Transwell^®^ insert coated with the Matrigel (CytoSelect 24-well Cell Invasion Assay Kit; Cell Biolabs, CA, USA), and cultured for 48 h with the complete medium in the lower chamber. The migration assays were performed using a similar procedure without coating the filters with Matrigel. All cells were fixed with ethanol (Aladdin, Shanghai, China), stained with crystal violet (Beyotime Biotechnology, Shanghai, China), and counted by Image J software (National Institutes of Health, USA). Each experiment was repeated three times.

**1.3 Animal experiments (supplementary)**

Three types of murine models were established in this article and were used to complete the experiments of nine panels.

(1) Figure S1E, Supporting Information (the xenograft model): the mice (n = 18) were randomly divided into three groups: the PM_2.5_-0 μg/mL group (treated with HBE cells exposed to PM_2.5_ at a concentration of 0 μg/mL for 30 generations); the PM_2.5_-10 μg/mL group (treated with HBE cells exposed to PM_2.5_ [10 μg/mL]); and the PM_2.5_-50 μg/mL group (treated with HBE cells exposed to PM_2.5_ [50 μg/mL]). The mice were injected subcutaneously into their left dorsal flanks with 5 × 10^6^ cells/0.1 mL/mouse on day 1.

(2) Figure S1F, Supporting Information (the hepatic metastasis model): the mice (n = 18) were divided similarly to (1), except that the injection method was changed to intrasplenically injected with 0.1 mL/mouse of a cell suspension containing 1 × 10^6^ cells on day 1.

(3) Figure S1G, Supporting Information (the lung metastasis model): the mice (n = 18) were grouped similarly to (1), but the injection method was changed to tail vein injection with 0.1 mL/mouse of a cell suspension containing 1 × 10^6^ cells on day 1.

(4) Figure 2D (the xenograft model): the mice (n = 24) were randomly divided into four groups: the PM_2.5_ (0 μg/mL) + NC group (treated with the “PM_2.5_ [0 μg/mL] + NC” stable cell lines); the PM_2.5_ (0 μg/mL) + circDNA2 KD group (treated with the “PM_2.5_ [0 μg/mL] + circDNA2 KD” stable cell lines); the PM_2.5_ (50 μg/mL) + NC group (treated with the “PM_2.5_ [50 μg/mL] + NC” stable cell lines); and the PM_2.5_ (50 μg/mL) + circDNA2 KD group (treated with the “PM_2.5_ [50 μg/mL] + circDNA2 KD” stable cell lines). The mice were injected with the same method and number of cells in (1).

(5) Figure 2E (the hepatic metastasis model): the mice (n = 24) were divided similarly to (4) and injected with the same method and number of cells in (2).

(6) Figure 2F (the lung metastasis model): the mice (n = 24) were divided similarly to (4) and injected with the same method and number of cells in (3).

(7) Figure 3K (the xenograft model): the mice (n = 18) were randomly divided into three groups: the NC group (treated with the “PM_2.5_ [50 μg/mL] + NC” stable cell lines); the circDNA2 OEX group (treated with the “circDNA2 OEX” stable cell lines); and the circDNA2/GADD45A OEX group (treated with the “circDNA2/GADD45A OEX” stable cell lines). The mice were injected with the same method and number of cells in (1).

(8) Figure 3L (the hepatic metastasis model): the mice (n = 18) were divided similarly to (7) and injected with the same method and number of cells in (2).

(9) Figure 3M (the lung metastasis model): the mice (n = 18) were divided similarly to (7) and injected with the same method and number of cells in (3).

**1.****4 Western blotting (WB) assay**

WB assays were conducted as previously showed [1]. Total proteins were extracted using the RIPA lysis buffer containing phenylmethylsulfonyl fluoride (PMSF) and protease inhibitor mixture (Cat: 89900, Thermo Fisher Scientific, CA, USA), and the protein concentrations were measured by Pierce^TM^ BCA Protein Assay Kit (Cat: 23227, Thermo Fisher Scientific). Extracted protein samples together with appropriate protein ladders were loaded in electrophoretic lanes. After electrophoresis, the protein was transferred onto a polyvinylidene difluoride (PVDF; Merck KGaA, Darmstadt, Germany) membrane, blocked at room temperature (RT) for 1 h, and incubated with the following primary antibodies: anti-YTHDF2 (Cat: 24744-1-AP, Proteintech, Hubei, China), anti-GADD45A (Cat: TA376458, OriGene, Jiangsu, China), anti-β-actin (Cat: 66009-1-Ig, Proteintech).

**1.5 Extraction of circRNA**

The total RNA was extracted using the TRIzol reagent (Thermo Fisher Scientific). Nucleocytoplasmic separation experiment was performed with the PARIS^TM^ Kit (Cat: AM1921, Thermo Fisher Scientific). For circRNA amplification, the total RNA was incubated for 15 min at 37°C with 3 U/mg Purelink RNase R (Thermo Fisher Scientific) and subsequently purified with RNeasy MinElute Cleaning Kit (Qiagen, Hilden, Germany), according to the manufacturer’s protocols.

**1.6 Quantitative real-time reverse transcription polymerase chain reaction (qRT-PCR)**

TRIzol reagent (Thermo Fisher Scientific) was used to isolate total RNA, and the cDNA was synthesized using the ReverTra Ace^®^ qPCR RT Kit (Cat: FSQ-101, Toyobo Co., Ltd., Osaka, Japan) from 500 ng RNA with random primers. The expression levels of circRNA and mRNA were determined using qRT-PCR with SYBR^®^ Green Realtime PCR Master Mix- Plus (Cat: QPK-211, Toyobo Co., Ltd.) according to the manufacturer’s instructions, and were normalized to β-actin, U6 and GAPDH. Each experiment was carried out in triplicates. The primer sequences used in the experiment were shown in Table S1.

**1.7 Dual-luciferase report assay**

Cells were seeded in 12-well plates (Corning Incorporated) in triplicates. Lipofectamine 2000 reagent (Thermo Fisher Scientific) was then used to co-transfect the psiCHECK reporter plasmids fused with either wild-type or mutant GADD45A. Finally, the Dual-Luciferase^®^ Reporter Assay System (Cat: E1090, Promega Corporation, WI, USA) was used to analyze Fluc and Rluc luciferase activities after 48 h.

**1.8 RNA stability assay**

HBE cells were cultured with Actinomycin D (2 μg/mL; Merck KGaA). After incubation, the circDNA2 level was detected by qRT-PCR.

**1.9** **Tissue microarray (TMA) construction and In situ hybridization (ISH)/Immunohistochemistry (IHC) analysis**

The lung cancer TMAs were purchased from Shanghai Outdo Biotech (Cat. HLugA180Su11). The slide consisted of 180 cores, which included 90 pairs of lung cancer and paired adjacent non-tumor tissues, and 86 cases diagnosed as lung adenocarcinoma. The study was conducted after the approval of the Shanghai Outdo Biotech Company Ethics Committee (approval number: SHYJS-CP-2206001). The clinical samples were obtained from patients and healthy volunteers after providing written informed consent, and their detailed information has been shown in Table S2. For circDNA2 detection assay, the detection was conducted using the Enhanced Sensitive ISH Detection kit I (Cat: MK1034, BOSTER, Hubei, China), following the manufacturer’s instructions. Briefly, the tissues were incubated with the specific digoxin-labeled circDNA2 probe. After blocking, the expression of circDNA2 was visualized by DAB staining. For the GADD45A detection IHC assay, the slices were coupled with anti-GADD45A antibody (Cat: TA376458, OriGene) antibodies at 4°C overnight. After staining with DAB (Zhongshan Biotech, Beijing, China), the samples were finally evaluated as previously established [2].

**1.10 MS2-tagged pull-down assay**

As described elsewhere [3], HBE cells were co-transfected with circDNA2-MS2 and Flag-MS2 OEX plasmid (constructed by Genechem, Shanghai, China) using Lipofectamine 2000 (Thermo Fisher Scientific). After 48 h, the proteins were pulled down using Flag-tag Protein IP Assay Kit（Cat: P2181S, Beyotime Biotechnology）and stained by Silver dye. Finally, the pulled proteins were further analyzed by WB or mass spectrometry (MS).

**1.11 RNA pull-down analysis**

As described in our previous research [4], biotin-labeled RNA for liner sequence of circDNA2 was generated by an *in vitro* transcription reaction with the Biotin RNA Labeling Mix (Roche, Mannheim, Germany) and T7 RNA polymerase (Roche), and then treated with RNase-free DNase I (Takara Shuzo, Kyoto, Japan). After incubation with guide oligonucleotide targeting circular junction, the liner probe was then circularized using T4 RNA ligase I, treated with RNase R. After purified with RNeasy Mini Kit (Qiagen), the biotin-labeled RNA probe (3 μg) was then incubated with cell extracts from HBE cells at RT for 2 h, and treated with 35 μL of Streptavidin C1 magnetic beads (Thermo Fisher Scientific) for 1 h. After washed, the retrieved protein was detected by WB.

Supplementary Table S1. The primer sequences used in qRT-PCR assay

|  | Forward (5’-3’) | Reverse (5’-3’) |
| --- | --- | --- |
| circDNA2 | GCTAATGCCTGCTTCGGAAA | ATCTAGATGGTTGGCAGGCA |
| circABCA3 | CAGACCCCTTCCTCGTGG | ATGGTCACCGTCAGTCTCTG |
| circCORO1C | AGGTCTCAGGCTCTTCAGGA | CAGAGCAAGTTTCGGCATGT |
| circCDK10 | CCTGAGCCTGGAAACCCA | CGGTAACCGTATCTCCCTC |
| circRPH1 | CTGAGACTAGGGCCAGAGG | GGAGTGGAGTGACAGGACG |
| GADD45A | CTGGAGGAAGTGCTCAGCAAAG | AGAGCCACATCTCTGTCGTCGT |
| GADD45B | GCCAGGATCGCCTCACAGTGG | GGATTTGCAGGGCGATGTCATC |
| DNA2 | AGAGCTGTCCTGAGTGAAACT | GAAACACCTCATGGAGAACCG |
| β-actin | CATGTACGTTGCTATCCAGGC | CTCCTTAATGTCACGCACGAT |
| U6 | CTCGCTTCGGCAGCACATATACT | ACGCTTCACGAATTTGCGTGTC |

Supplementary Table S2. Correlation between circDNA2/GADD45A expression and clinicopathological characteristics

|  | variables | circDNA2 expression | | p value | GADD45A expression | | p value |
| --- | --- | --- | --- | --- | --- | --- | --- |
|  |  | low | high |  | low | high |  |
| Age (year) |  |  |  | 0.5315 |  |  | 0.8233 |
|  | ≤60 | 25 | 19 |  | 17 | 24 |  |
|  | ＞60 | 20 | 20 |  | 18 | 23 |  |
| Sex |  |  |  | 0.9625 |  |  | 0.3544 |
|  | Male | 24 | 21 |  | 20 | 22 |  |
|  | Female | 21 | 18 |  | 15 | 25 |  |
| TNM stage |  |  |  | 0.0253 |  |  | 0.3368 |
|  | Ι/II | 40 | 27 |  | 26 | 39 |  |
|  | III | 5 | 12 |  | 9 | 8 |  |

**Supplementary Figure Legends**

**Figure S1. Long-term PM_2.5_ exposure promotes the malignant transformation of HBE cells**

A). Schematic overview of the chronic PM_2.5_ exposure model. B-D). Effects of PM_2.5_ exposure on the colony formation B), migration ability C), and invasion ability D) of HBE cells *in vitro* (n = 6 per group, compared with the PM_2.5_ [0 µg/mL] group, one-way ANOVA, error bars represent SD, duplicate measurement per replicate). E-G). Effects of PM_2.5_ exposure on the HBE flank tumor burden E), and hepatic F), or lung G) metastasis *in vivo* (n = 6 per group, duplicate measurement per replicate). ^***^*P* < 0.001. Representative staining images are shown, and the scale bars are marked in each image.


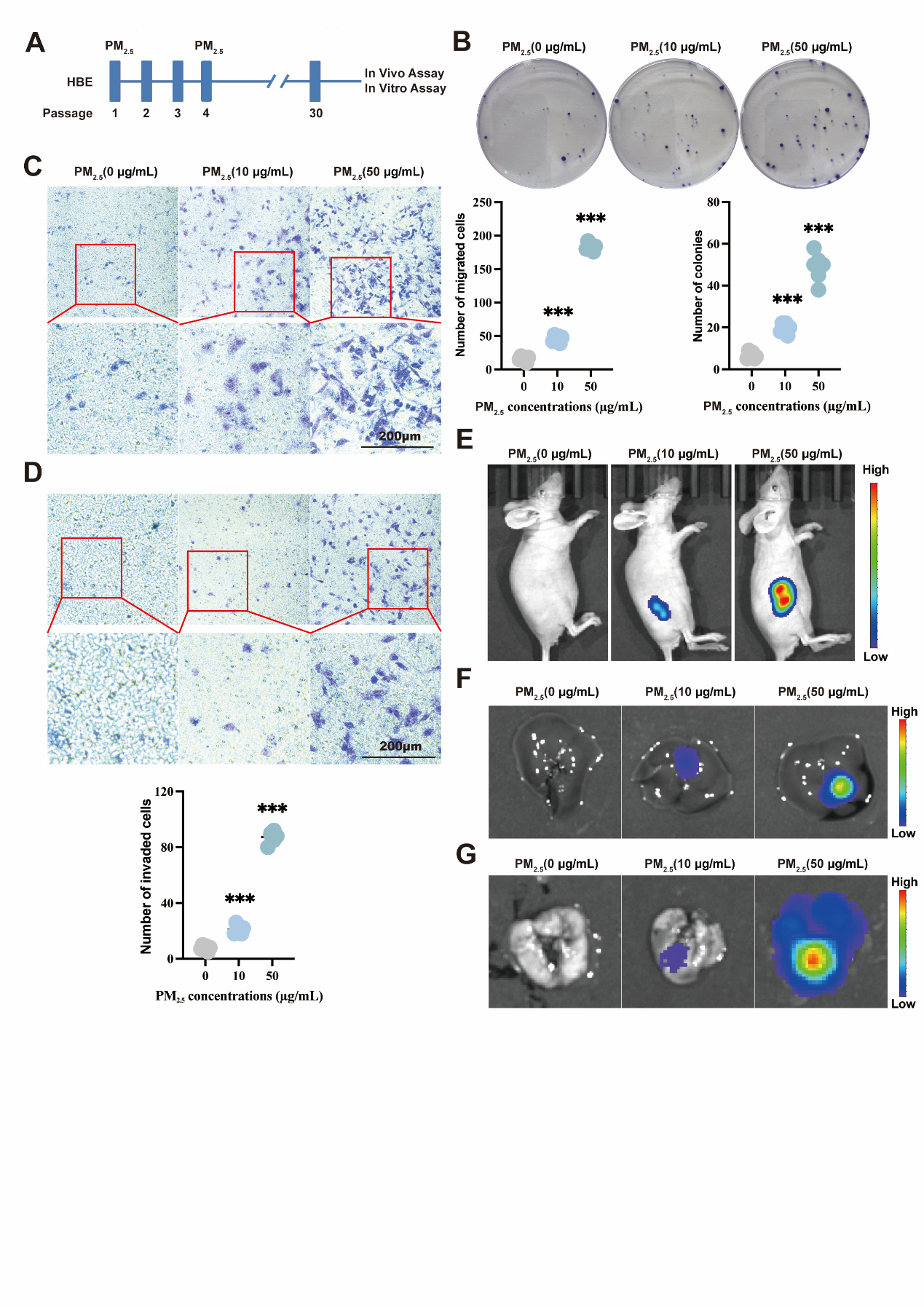


**Figure S2. Expression of four indicated circRNAs in long-term PM_2.5_ exposure HBE cells (n = 3 per group, compared with the PM_2.5_ [0 µg/mL] group, one-way ANOVA, error bars represent SD, duplicate measurement per replicate). ns: no significant difference, ^*^*P* < 0.05, ^**^*P* < 0.01.**

**
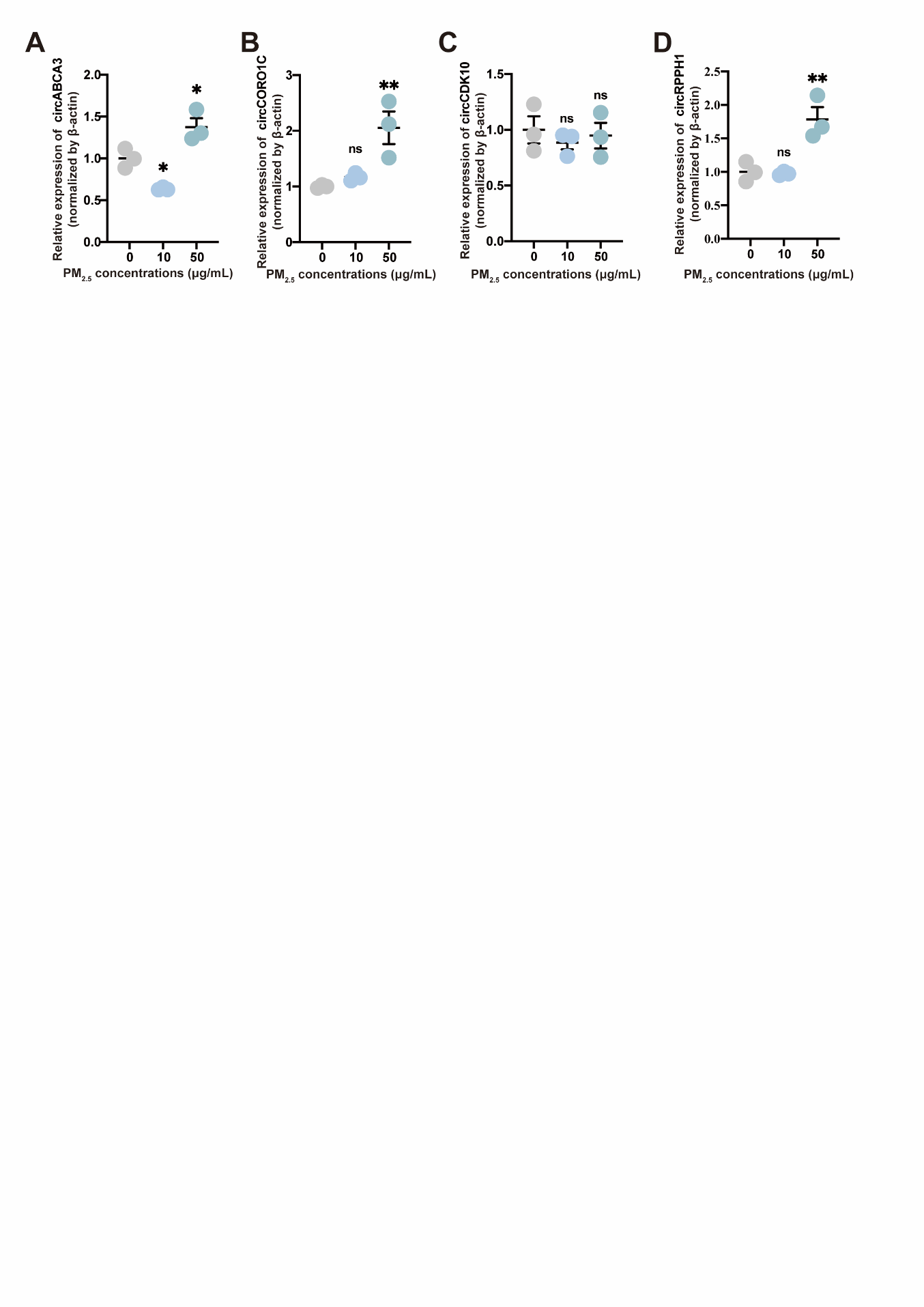
**

**Figure S3. The mRNA expression level of circDNA2 in PM_2.5_-transformed HBE cells (n = 3 per group, compared with the PM_2.5_ [0 µg/mL] NC group, one-way ANOVA,** **error bars represent SD, duplicate measurement per replicate). ^*^*P* < 0.05, ^**^*P* < 0.01, ^***^*P* < 0.001.**

**
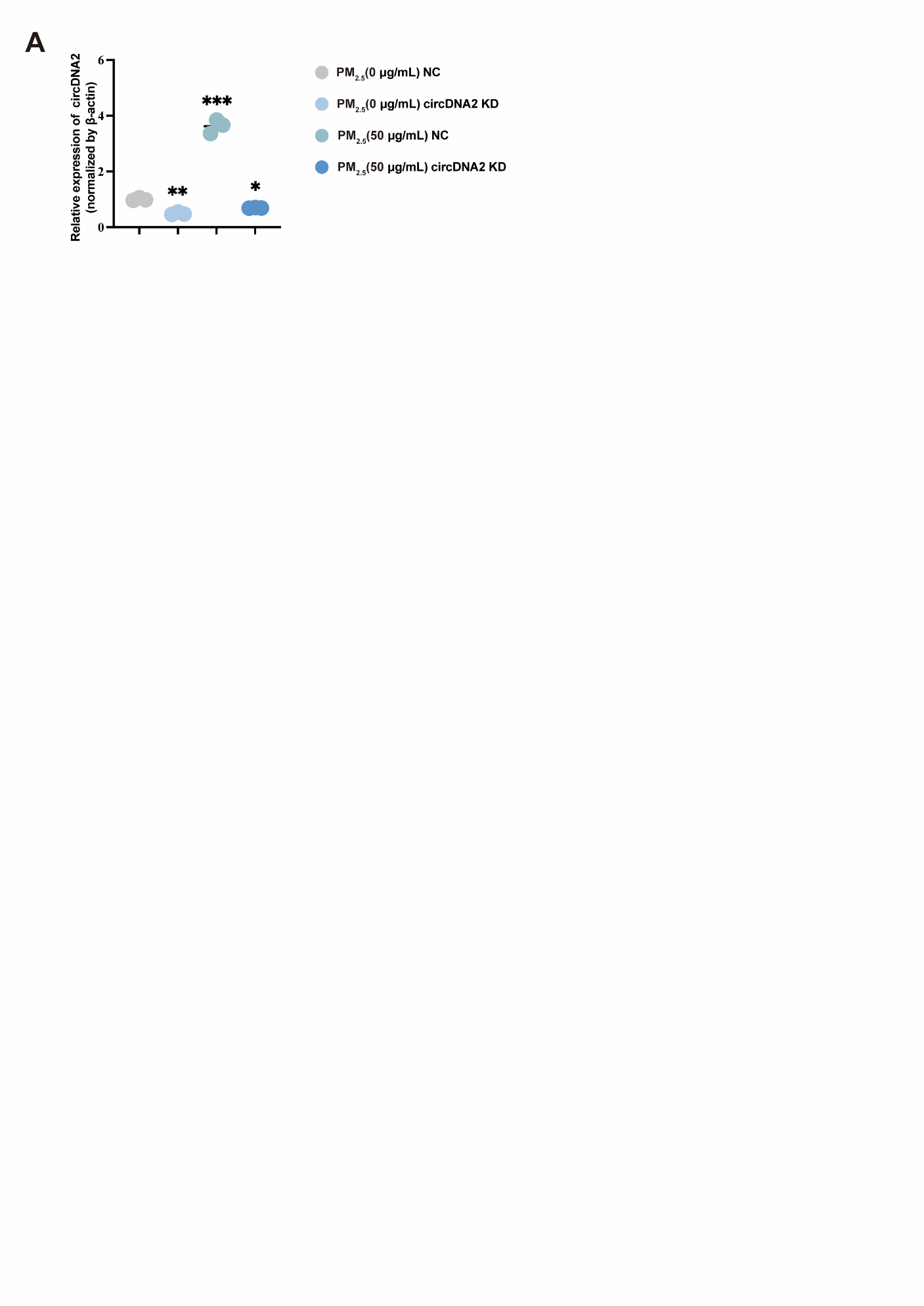
**

**Figure S4. Effects of circDNA2 and/or GADD45A OEX on the PM_2.5_-transformed HBE cells *in vitro***

A). The mRNA levels of circDNA2 and GADD45A in PM_2.5_-transformed HBE cells (n = 3 per group, NC: vehicle control, compared with NC, one-way ANOVA, error bars represent SD, duplicate measurement per replicate). B).Effects of circDNA2 and/or GADD45A OEX on the colony formation, migration ability, and invasion ability of PM_2.5_ (50 μg/mL)-transformed HBE cells *in vitro* (n = 6 per group, compared with the PM_2.5_ [50 µg/mL]-transformed HBE cells treated NC lentivirus, one-way ANOVA, error bars represent SD, duplicate measurement per replicate). ns: no significant difference, ^**^*P* < 0.01, ^***^*P* < 0.001.

**
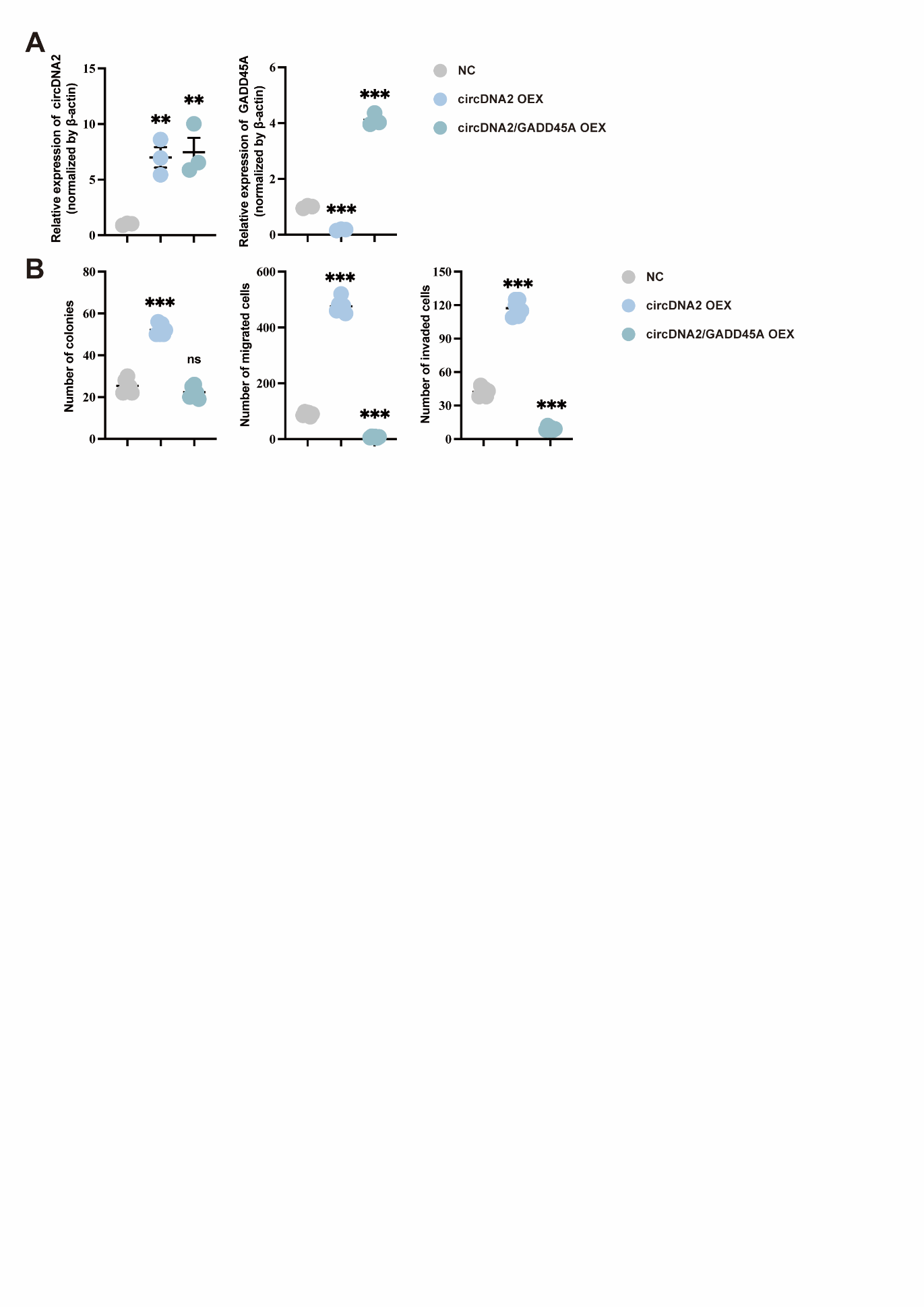
**

**Figure S5. Three other YTHDF2** **peptides identified in circDNA2-enriched proteins.**

**
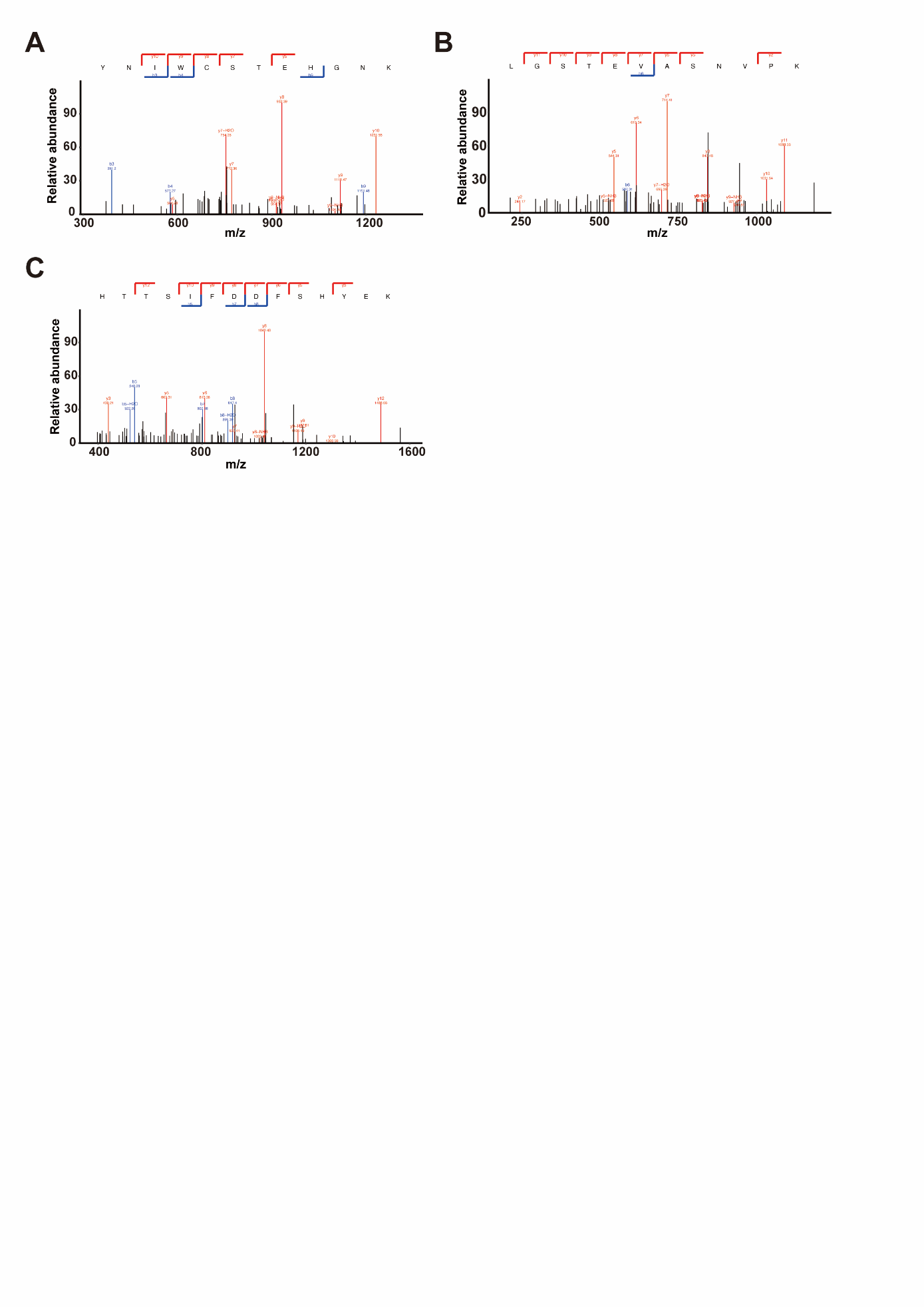
**

**Figure S6. GADD45A is a potential prognostic biomarker for lung cancer**

A). The expression levels of GADD45A in TMAs were evaluated by IHC, and the differences in staining scores between lung cancer lesions and adjacent tissues are shown. B). KM curves depicting the overall survival of patients with lung cancer. C). Multivariate Cox regression analysis of patients with lung cancer.


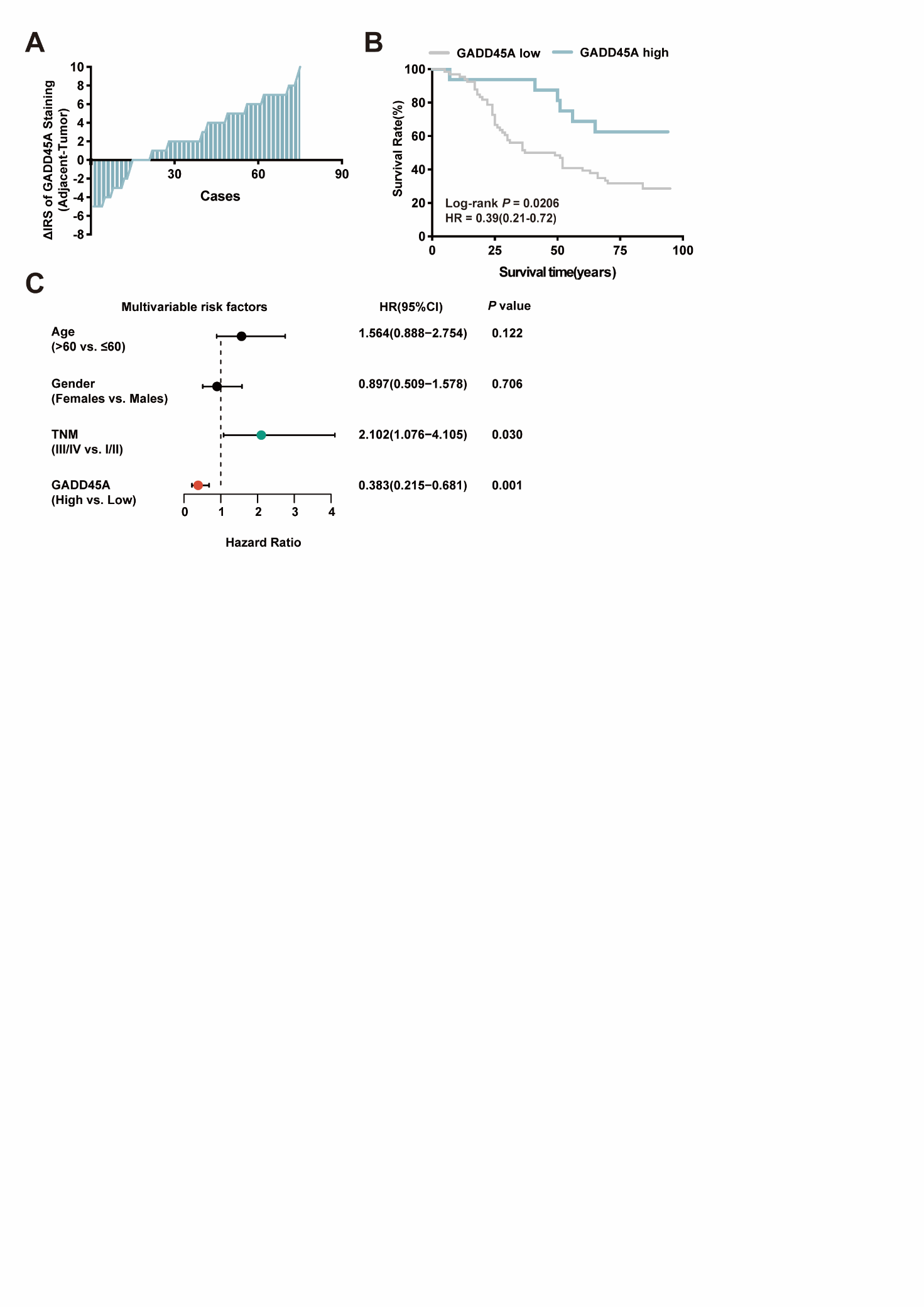


**References**

[1] R. S. Akchurin, A. A. Beliaev, G. N. Zmievskoi, S. E. Ragimov, *Kardiologiia* **1989**, *29* (6), 117.

[2] S. Wu, J. Yun, W. Tang, G. Familiari, M. Relucenti, J. Wu, X. Li, H. Chen, R. Chen, *ACS Nano* **2023**, *17* (12), 11838, https://doi.org/10.1021/acsnano.3c03050.

[3] T. Xu, M. Xiong, Q. Hong, B. Pan, M. Xu, Y. Wang, Y. Sun, H. Sun, Y. Pan, S. Wang, B. He, *Cell Death Dis* **2024**, *15* (2), 153, https://doi.org/10.1038/s41419-024-06527-7.

[4] H. Yang, X. Li, Q. Meng, H. Sun, S. Wu, W. Hu, G. Liu, X. Li, Y. Yang, R. Chen, *Mol Cancer* **2020**, *19* (1), 13, https://doi.org/10.1186/s12943-020-1139-3.
